# Contribution of the Unified Health Care System to mammography screening in Brazil, 2013*

**DOI:** 10.1590/0100-3984.2014.0129

**Published:** 2016

**Authors:** Ruffo Freitas-Junior, Danielle Cristina Netto Rodrigues, Rosangela da Silveira Corrêa, João Emílio Peixoto, Humberto Vinícius Carrijo Guimarães de Oliveira, Rosemar Macedo Sousa Rahal

**Affiliations:** 1 PhD, Professor and Coordinator of the Breast Disease Program at the Faculdade de Medicina da Universidade Federal de Goiás (UFG), Goiânia, GO, Brazil.; 2 PhD, Psychologist, Member of the Goiânia Breast Disease Research Network and Breast Disease Program at the Faculdade de Medicina da Universidade Federal de Goiás (UFG), Goiânia, GO, Brazil.; 3 PhD, Senior Technologist for the Comissão Nacional de Energia Nuclear/Centro Regional de Ciências Nucleares do Centro-Oeste, Goiânia, GO, Brazil.; 4 PhD, Medical Physicist, Consultant for the Ionizing Radiation Quality Control Sector of the Instituto Nacional de Câncer (INCA), Rio de Janeiro, RJ, Brazil.; 5 Graduate Student in Environmental Sciences at the Universidade Federal de Goiás (UFG), Goiânia, GO, Brazil.; 6 PhD, Professor at the Faculdade de Medicina da Universidade Federal de Goiás (UFG), Goiânia, GO, Brazil.

**Keywords:** Breast neoplasms/prevention & control, Mass screening/statistics & numerical data, Mammography/statistics & numerical data, Health services/statistics & numerical data

## Abstract

**Objective:**

To estimate the coverage of opportunistic mammography screening performed via the Brazilian Sistema Único de Saúde (SUS, Unified Health Care System), at the state and regional level, in 2013.

**Materials and Methods:**

This was an ecological study in which coverage was estimated by determining the ratio between the number of mammograms performed and the expected number of mammograms among the population of females between 50 and 69 years of age. The number of mammograms performed in the target population was obtained from the Outpatient Database of the Information Technology Department of the SUS. To calculate the expected number of mammograms, we considered 58.9% of the target population, the proportion that would be expected on the basis of the recommendations of the Brazilian National Cancer Institute.

**Results:**

In 2013, the estimated national coverage of mammography screening via the SUS was 24.8%. The mammography rate ranged from 12.0% in the northern region to 31.3% in the southern region. When stratified by state, coverage was lowest in the state of Pará and highest in the state of Santa Catarina (7.5% and 35.7%, respectively).

**Conclusion:**

The coverage of mammography screening performed via the SUS is low. There is a significant disparity among the Brazilian states (including the Federal District of Brasília) and among regions, being higher in the south/southeast and lower in the north/northeast.

## Abstract (pt)

**Objetivo:**

Estimar a cobertura mamográfica em rastreamento oportunista realizado pelo Sistema Único de Saúde (SUS) no Brasil, nas suas regiões e nas Unidades da Federação, no ano de 2013.

**Materiais e Métodos:**

Estudo ecológico, em que a estimativa da cobertura foi a proporção entre o número de exames realizados e o número de exames esperados na população feminina na faixa etária de 50 a 69 anos. O número de exames realizados refere-se aos dados da produção mamográfica da população alvo, disponíveis no Sistema de Informações Ambulatoriais do Departamento de Informática do SUS. Para o cálculo do número de exames esperados considerou-se 58,9% da população alvo, tendo em vista as recomendações do Instituto Nacional de Câncer.

**Resultados:**

A estimativa da cobertura mamográfica no rastreamento realizado pelo SUS no Brasil, em 2013, foi 24,8%. A prevalência de mamografias variou de 12,0% na Região Norte a 31,3% na Região Sul. Ao estratificar por Unidades da Federação, a menor cobertura foi no Pará (7,5%) e a maior cobertura foi em Santa Catarina (35,7%).

**Conclusão:**

A cobertura mamográfica do SUS no Brasil é baixa. Existe uma desigualdade significativa entre as Unidades da Federação, sendo maior nas regiões Sul e Sudeste e menor nas regiões Norte e Nordeste do país.

## INTRODUCTION

Recent studies carried out in various countries have noted a reduction in the rate of mortality due to breast cancer as a result of the actions of secondary prevention and treatment advances that have a direct influence on the survival of patients with this disease^(1-6)^.

It should be emphasized that the data in the literature are robust with regard to the importance of mammography as a method of screening for breast cancer. In the 1970s and 1980s, randomized studies conducted in developed countries evaluated the implementation of mass screening programs and proved the effectiveness of mammography screening as one of the factors responsible for reducing the breast cancer mortality rate^(7-13)^.

In Brazil, access to mammography is via the *Sistema Único de Saúde* (SUS, Unified Health Care System) or the National Health Insurance Agency, by direct negotiation between the individual and the health care facility. The SUS is the official government system and was created in order to uphold the Brazilian constitution, which states that health care is a right of all citizens and a duty of the State^(14-17)^.

The relevance of this system in Brazil is unquestionable, given that more than 70% of the Brazilian population depends solely on the SUS for access to health care services^(15-17)^. In recent years, public policies governing the SUS have advanced regarding the control of breast cancer, and governmental strategies have improved its early detection^(18-20)^.

Although there has been a reduction in the number of breast cancer cases detected at advanced stages in Brazil, there is still a worrisome number of such cases, calling for the implementation of policies regarding mammography screening that are pertinent to the peculiarities of the country^(21,22)^. Given that Brazil is a country of continental dimensions^(23)^, knowledge of mammography coverage could contribute to improving the effectiveness of public policies in the country.

The aim of this study was to estimate the mammography coverage in women 50-69 years of age, as well as to describe the distribution of women, mammogram production and the proportion of tests performed, by age group, in breast cancer screening via the SUS in Brazil, by region and by state, in 2013.

## MATERIALS AND METHODS

This was an ecological study in which the data regarding mammograms performed via the SUS in Brazil were analyzed, by region and by state, for the year 2013.

Brazil has a landmass of 8,515,767.049 km^2^. As of 2013, there were 201,032,714 inhabitants, distributed among 27 Federal Units (FUs) or states: 26 states and the Federal District of Brasília. These FUs are distributed among five regions^(24)^: north, northeast, southeast, south, and centralwest.

The northern region encompasses an area of 3,869,637.9 km^2^ and comprises the states of Acre, Amapá, Amazonas, Pará, Rondônia, Roraima, and Tocantins, with 16,983,484 inhabitants, collectively, in 2013. The northeastern region includes the states of Maranhão, Piauí, Ceará, Rio Grande do Norte, Paraíba, Pernambuco, Alagoas, Sergipe, and Bahia, with a collective population of 55,794,707 inhabitants in 2013, encompassing a total area of 1,561,177.8 km^2(24)^. The southeastern region comprises the states of Espírito Santo, Minas Gerais, Rio de Janeiro, and São Paulo, with 84,465,570 inhabitants, collectively, encompassing a total area of 927,286.2 km^2^. The southern region, which includes the states of Paraná, Santa Catarina, and Rio Grande do Sul, covers a total of 577,214.0 km^2^, with a collective population of 28,795,762 inhabitants^(24)^. Finally, the central-west region, comprising the states of Mato Grosso, Mato Grosso do Sul, and Goiás, together with the Federal District of Brasília, occupies 1,580,451.15 km^2^ and had population of 14,993,191 in 2013^(24)^.

### Target population

The target population was that of women between the 50 and 69 years of age. Data were obtained from the *Departamento de Informática do SUS* (DATASUS, Information Technology Department of the SUS) Database of Demographic and Socioeconomic Characteristics^(24)^.

### Estimated coverage

To estimate the degree of coverage, we calculated the biennial screening rate needed in order to reach 100% of the target population. That indicator was expressed as a percentage and was based on the ratio between the number of examinations carried out and the number of examinations expected in the target population^(25)^.

To calculate the expected number of mammograms, we considered 58.9% of the target population, the proportion that would be expected on the basis of the recommendations of the Brazilian *Instituto Nacional de Câncer* (INCA, National Cancer Institute). In scheduling biennial screening procedures, in a given year, 50% of women between 50 and 69 years of age will undergo clinical examination of the breast and the other 50% will undergo clinical breast examination and mammography. It is expected that 8.9% of the women who undergo clinical examination of the breast only will subsequently require diagnostic mammography^(26)^.

To calculate the number of mammograms performed, we obtained production data from the Outpatient Database of the DATASUS^(27)^, which is the system used by the Brazilian National Ministry of Health to monitor such examinations.

The attributes for data collection on the DATASUS website (http://www2.datasus.gov.br/DATASUS/index.php?area=0701&item=1&acao=22&pad=31655) were as follows: → Health Database → Health Care → Outpatient Production by Place of Residence (since 2008) → National, Regional, and State → Number of Examinations Approved for Payment → Procedures (Mammography - code 0204030030 and Bilateral Screening Mammography - code 0204030188) → Female → Age Group, 50-69 years.

It is worth mentioning that, as in the calculation of the number of examinations expected, in which the proportion of screening mammograms (50%) and the proportion of diagnostic mammograms (8.9%) were summed, the same procedure was adopted for the calculation of the number of scans performed, in which the numbers of examinations with the codes 0204030030 and 0204030188 were totaled.

### Statistical analysis

The data collected were subjected to statistical analysis in order to determine whether there was proportional similarity between the number of examinations carried out and the number of examinations expected in the target population, by regions, by FUs, and nationwide. For that, we used the Descartes' Rule of Signs statistical test, employing the Statistical Package for the Social Sciences, version 17.0 (SPSS Inc., Chicago, IL, USA).

## RESULTS

In 2013, the female population of Brazil was 101,695,856, accounting for 51% of the general population. Of those, 8,359,536 (8.2%) resided in the northern region, 28,388,309 (27.9%) resided in the northeastern region, 42,881,344 (42.2%) resided in the southeastern region, 14,548,385 (14.3%) resided in the southern region, and 7,518,282 (7.4%) resided in the central-west region.

Evaluating the distribution of the women by age group, we found that 64.5% were under 40 years of age, 13.0% were 40-49 years of age, 10.4% were 50-59 years of age, 6.6% were 60-69 years of age, and 5.5% were over 69 years of age.

The proportion of women under 40 years of age was highest in northern region, whereas those of women 40-49, 50-59, and 60-69 years of age were highest in the southern region. The proportion of women over 69 years of age was highest in the southern and southeastern regions. The proportion of women in the age bracket for which the INCA recommends breast cancer screening (50-69 years of age) was highest (19.6%) in the southern region, followed by the southeastern, central-west, northeastern, and northern regions (19.1%, 15.3%, 14.6%, and 11.2%, respectively).

In 2013, the SUS approved payment for 4,663,784 mammograms performed in Brazil. Of those, 4,659,162 (99.9%) were performed in women. Of the mammograms performed in women, 207,375 (4.5%) were in women under 40 years of age, 1,632,131 (35.0%) were in women 40- 49 years of age, 2,523,639 (54.2%) were in women 50-69 years of age, and 296,017 (6.4%) were in women over 69 years of age.

Among women in the age bracket for which the INCA recommends biennial breast cancer screening (50-69 years of age), the number of mammograms expected in 2013 was 10,174,905. However, the number performed in the target population was 2,525,281, which allows us to estimate a coverage of 24.8% among women between 50 and 69 years of age in Brazil. The estimated coverage, by region and by FU, is presented in Table 1. As can be seen in the table, the mammography rate ranged from 12.0% in the north to 31.3% in the south. In the target population (women 50-69 years of age), the ratio of mammograms performed to mammograms expected was comparable among the five regions (*p* = 0.063).

**Table 1:** Coverage of opportunist mammography screening in women 50–69 years of age in Brazil, by region and by Federal Unit, in 2013.

Region	Number of mammograms expected*	Number of mammograms performed^†^	Coverage (%)
Federal Unit			
Federal Unit			
North	552,437	66,394	12.0
Rondônia	63,737	5,267	8.3
Acre	22,954	2,620	11.4
Amazonas	113,423	24,764	21.8
Roraima	14,004	1,142	8.2
Pará	266,046	20,062	7.5
Amapá	19,392	1,546	8.0
Tocantins	52,882	10,993	20.8
Northeast	2,445,168	541,421	22.1
Maranhão	241,192	22,944	9.5
Piauí	142,835	24,870	17.4
Ceará	390,476	52,867	13.5
Rio Grande do Norte	154,113	26,598	17.3
Paraíba	184,253	37,752	20.5
Pernambuco	437,516	129,464	29.6
Alagoas	135,597	35,925	26.5
Sergipe	91,346	17,570	19.2
Bahia	667,840	193,431	29.0
Southeast	4,821,591	1,292,964	26.8
Minas Gerais	1,110,283	395,126	35.6
Espírito Santo	194,560	48,157	24.8
Rio de Janeiro	1,034,567	150,994	14.6
São Paulo	2,482,181	698,687	28.1
South	1,674,653	524,412	31.3
Paraná	601,156	195,928	32.6
Santa Catarina	358,815	128,006	35.7
Rio Grande do Sul	714,682	200,478	28.1
Central-west	681,055	100,090	14.7
Mato Grosso do Sul	122,341	22,389	18.3
Mato Grosso	130,464	15,415	11.8
Goiás	299,025	43,941	14.7
Distrito Federal	129,226	18,345	14.2

Sources:

* DATASUS Database of Demographic and Socioeconomic Characteristics; and

† DATASUS Outpatient Database.

When the regional results were stratified by FU, the mammography rate in the northern region was found to range from 7.5%, in Pará, to 21.8%, in Amazonas, although there was no statistical similarity (*p* = 0.016). However, when the data for the states of Pará and Amapá were removed from the statistical analysis, the results were similar among the remaining FUs (*p* = 0.063).

In the northeastern region, the coverage ranged from 9.5%, in Maranhão, to 29.6%, in Pernambuco. As was observed for the northern region, there was no statistical similarity among the data for the northeastern region (*p* = 0.004). However, when the FUs were divided into two groups, by the estimated coverage, the variation in coverage was 11.1- 14.5% in the first group (*p* = 0.125) and 16.5-32.02% in the second (*p* = 0.063). Among the FUs within the northeastern region, the estimated coverage was highest for the state of Bahia and lowest for the state of Maranhão, being three times higher for the former than for the latter.

In the southeastern region, there was no significant difference among the FUs (*p* = 0.125). However, the estimated coverage was twice as high for the state of Minas Gerais than for the state of Rio de Janeiro.

In the southern region, there was considerable similarity among the FUs (*p* = 0.250). The estimated coverage was higher for the state of Santa Catarina than for any of the other FUs in that region, as well as being higher than that calculated for any other FU in the country.

The estimated coverage in the central-west region ranged from 10.3%, in the Federal District of Brasília, to 19.7%, in the state of Mato Grosso do Sul, with no significant difference among the FUs within the region (*p* = 0.125).

## DISCUSSION

Recent studies conducted in Brazil have highlighted the importance of imaging examinations, especially mammography, to improving the diagnosis of breast cancer^(28-34)^.

The implementation of breast cancer screening programs has been found to be justified in contexts of high incidence, contributing to a reduction in mortality by identifying the disease in early stages and improving its prognosis^(4,7,9,11)^.

The World Health Organization (WHO) has determined that, before a mass screening program can be implemented, at least 70% of the target population should have access to the examination^(35)^. For 2013, the estimated contribution of the SUS to mammography screening in Brazil was 24.8% overall and 7.5-35.7% among the FUs, which indicates uneven coverage that is far below that recommended by the WHO. That is in agreement with the data in the literature^(25,36)^.

Although management tools that facilitate the evaluation of the performance of the SUS in controlling breast cancer, such as the National Program for Quality Control in Mammography and the Breast Cancer Control Database^(19,37)^, have been implemented in Brazil, mammography screening in the country is still opportunistic, being limited by logistical and economic problems, as well as by sociocultural barriers^(38-40)^. However, there are some well-organized, although isolated, screening programs in the country, such as the one run by the Cancer Hospital of Barretos, in the state of São Paulo, which employs mobile units outfitted with mammography equipment in order to carry out active surveillance of the target population^(41,42)^.

Even without organized screening programs that cover all of the target population, the literature shows that the breast cancer mortality rate in Brazil began to stabilize after 1995. That stabilization could be related to improvement in the treatment of breast cancer and an increase of the human development index^(43,44)^, as well as to the opportunistic screening conducted via the SUS and complemented by the National Health Insurance Agency^(16,17,25)^.

However, there is still considerable disparity among the Brazilian population in terms of access to early detection, diagnosis, and treatment, whether via the SUS, upon which 70% of the population are dependent, the National Health Insurance Agency, or private health insurance companies^(16,17)^. Such disparity was seen in the present study, as evidenced by the differences in coverage, and in a study conducted by the Group for Breast Cancer Studies in Brazil^(21)^, which showed that 16.2% of the population treated at private hospitals had advanced tumors (stage III or IV) at diagnosis, compared with 36.9% of the population served by the public sector. The authors of that study found that mortality from stage III breast cancer was approximately 10% lower among individuals treated at public hospitals than among those treated at private hospitals^(21)^.

Factors such as cultural differences, socioeconomic differences, geographical location, and limited information on the part of the population can also impede access to health care services, thus increasing disparity^(41,45)^. The low rates of mammography coverage found in the present study underscore the need to research the geographic distribution of the mammography equipment available via the SUS and the influence that it can have on access to the test^(16,42)^.

There is as yet no consensus regarding the age at which the first mammogram should be performed or regarding the ideal frequency of mammography screening^(1-3,26,35,36)^. In the present study, the production data reveal that approximately 40% of all mammograms were conducted in women younger than the 50 years of age recommended by the INCA for the first mammogram^(26)^. Nevertheless, these results are in agreement with Federal Law No. 11,664 (signed on April 29, 2008), as well as with the recommendations of the Brazilian Breast Disease Society, the Brazilian College of Radiology and Diagnostic Imaging and the Federation of Brazilian Societies of Gynecology and Obstetrics, all of which state that mammography screening should begin at 40 years of age^(38,46)^.

Estimates of the incidence of breast cancer in Brazil indicate 57,120 new cases in 2014, the highest rates being in the south and much of the southeast^(47)^. The results of our study suggest that the efforts of the SUS related to screening for breast cancer should be maintained and even intensified in the southern and southeastern regions. In the northern, northeastern, and central-west regions of Brazil, there is a need for more robust public policies that can effectively increase mammography coverage among women.

The limitations of this study were that we used secondary data and that we evaluated opportunistic mammography screening programs, in which there is no control over the population that undergoes the test.

## CONCLUSION

The coverage of SUS-sponsored mammography screening in Brazil is low, falling far short of that recommended by the WHO. There is significant disparity among the various FUs, coverage being highest in the southern and southeastern regions and lowest in the northern and northeastern regions. Our results allow us to infer that, in Brazil, mammography coverage decreases as patient ages increase.
